# Efficacy and Safety of Tofacitinib in Lichen Planopilaris: A Retrospective Series of 74 Patients

**DOI:** 10.34172/aim.33237

**Published:** 2025-01-01

**Authors:** Mehdi Goodarzi, Sahar Dadkhahfar, Faranak Yahyaei, Zahra Razzaghi, Hamideh Moravvej

**Affiliations:** ^1^Skin Research Center, Shahid Beheshti University of Medical Sciences, Tehran, Iran; ^2^Laser Application in Medical Sciences Research Center, Shahid Beheshti University of Medical Sciences, Tehran, Iran

**Keywords:** Alopecia, Autoimmune diseases, Janus kinase inhibitors, Lichen planus, Tofacitinib

## Abstract

**Background:**

Lichen planopilaris (LPP) is a rare, inflammatory condition leading to scarring alopecia, predominantly affecting middle-aged women. Traditional treatments have shown limited efficacy, highlighting the need for novel therapeutic approaches. Tofacitinib, a Janus kinase (JAK) inhibitor, has shown promise in treating various autoimmune diseases, including autoimmune dermatological disorders. This study aims to evaluate the efficacy of tofacitinib in treating patients with LPP.

**Methods:**

We conducted a retrospective, single-center observational study at Shohadaye Tajrish Hospital, reviewing records of 74 patients with biopsy-confirmed LPP who had extensive and treatment-resistant disease. Patients were treated with tofacitinib 5 mg twice daily for at least 16 weeks. Efficacy was assessed using the LPP Activity Index (LPPAI), and adverse events were monitored.

**Results:**

This study evaluated 74 patients with LPP, predominantly female (83.3%), with a mean age of 46.64±8.05 years. The mean LPPAI score significantly decreased from 4.61±1.26 before treatment to 1.73±1.68 after six months (*P*<0.0001). Response rates varied: 21.62% within 1‒3 months, 24.32% within 3‒6 months, 33.78% within 6‒12 months, and 8.10% within 12‒24 months, with 6.75% showing no response. Adverse effects included headache (8.10%), hyperlipidemia (2.70%), elevated liver enzymes (5.40%), nausea (6.75%), and high blood pressure (4.15%).

**Conclusion:**

Tofacitinib represents a promising treatment for LPP, providing significant improvement in disease activity for most patients. Further research is needed to refine treatment protocols, understand predictors of response, and address gender-specific adverse effects.

## Introduction

 Lichen planopilaris (LPP) is an inflammatory condition characterized by the progressive destruction of hair follicles, leading to scarring alopecia.^1^ As a variant of lichen planus, it primarily affects the scalp but can occasionally involve other body parts.^2^ LPP is more common in females and typically starts between the ages of 40 and 70, and it is rare in children.^3-6^ The etiology of LPP remains poorly understood, although it is thought to have an autoimmune component.^7^ Patients with LPP often experience significant psychological distress due to irreversible hair loss and scalp discomfort.^8^ The main treatment objectives are to prevent the advancement of hair loss, alleviate symptoms, and preserve the existing hair follicles.^9^ Traditional treatment options, including corticosteroids and immunosuppressive agents, have had limited success, underscoring the need for more effective clinical treatments.^9^

 Tofacitinib, a Janus kinase (JAK1,3) inhibitor, has emerged as a promising treatment for various autoimmune diseases due to its ability to impair the JAK-STAT signaling pathway, which communicates extracellular signals to the cell nucleus, thereby affecting DNA transcription.^10-12^ Its efficacy has been well-documented in conditions such as rheumatoid arthritis, psoriasis, and alopecia areata, and emerging evidence suggests potential benefits in treating other autoimmune dermatological disorders, including LPP.^13-15^ By inhibiting specific pathways involved in the inflammatory process, tofacitinib may offer a novel approach to managing LPP, providing relief from symptoms and possibly halting disease progression.^16-18^

 This retrospective study aims to investigate the efficacy of tofacitinib in patients with LPP who were referred to a tertiary referral center from 2020 to 2022. By analyzing patient records, treatment outcomes, and side effects, this study seeks to provide valuable insights into the potential role of tofacitinib in managing LPP. Our research findings will contribute to the growing body of literature on the use of JAK inhibitors in dermatology and may lead to improved treatment approaches for individuals dealing with this challenging condition.

## Materials and Methods

###  Study Design and Patients

 We performed a retrospective single-center observational analysis to investigate the efficacy of tofacitinib for patients with LPP at a tertiary referral center. This research was approved by the institutional ethical board. After obtaining informed consent from the patients, we reviewed the records of 74 patients with extensive and treatment-resistant LPP who were referred to our dermatology clinic.

 For efficacy evaluation, patients with biopsy-confirmed LPP were included in the study. The sample size was determined based on the availability of eligible patients during the study period. As LPP is a rare condition, identifying a larger cohort was not feasible. Comparable studies examining the use of tofacitinib in autoimmune dermatological conditions, such as alopecia areata and other lichen planus subtypes, typically include sample sizes of 30–100 patients. These benchmarks guided our decision, ensuring the study remained practical while providing meaningful insights. Although formal power calculations were not performed due to the retrospective nature of this study, the significant reduction in the LPP Activity Index (LPPAI) observed in our cohort indicates the adequacy of the sample size.

 We excluded patients aged under 18 years and over 55 years, those with concurrent alopecia areata, active malignancy or history of malignancy in the past five years, active infection, pregnancy, and lactation, those using systemic medications such as systemic steroids, cyclosporine, hydroxychloroquine, and mycophenolate mofetil in the last three months, those with allergy to tofacitinib or any component of the drug formulation, latent tuberculosis, hepatitis B or C, HIV infection, and those diagnosed with bone marrow suppression (hemoglobin less than 9 g/dL, neutrophils less than 1000 cells/mm³, and lymphocytes less than 500 cells/mm³).

###  Data Collection

 All data on patient demographics were retrieved from patients’ medical records. Data were collected on age, sex, history of accompanying skin disease, history of autoimmune disease, family history of LPP or any autoimmune disease, drugs used in the past for this disease, and the response to previous treatments.

###  Outcomes and Definitions

 The patients underwent treatment with tofacitinib 5 mg twice a day for a minimum of 16 weeks. The dosage of TOF was tapered and then discontinued based on the patient’s condition. The patients were visited monthly and later every two months. Lab test monitoring was performed every two months. After treatment discontinuation, patients were followed up monthly for up to 6 months to assess the recurrence and any potential long-term side effects.

###  Statistical Analysis

 Data analysis was conducted using SPSS version 26.0 (IBM Corp., Armonk, NY, USA). Categorical variables were described with frequencies and percentages, while continuous variables were presented as means ± standard deviations. Normality was evaluated with Shapiro-Wilks test. To compare the LPPAI index before and after the study, Wilcoxon signed rank test was used. A significance level of *P*< 0.05 was considered in this study.

## Results

 This study evaluated 74 patients with a mean age of 46.6 ± 8.1 years, of whom 12 (16.2%) were male and 62 (83.8%) were female. The mean age at diagnosis was 42.0 ± 7.9 years, and the mean duration of disease was 55.0 ± 37.5 months.

 The median (IQR) LPPAI score before treatment was 5.6 (0.65), which decreased to 2.3 (2.17) at 6 months after treatment (*P *value = 0.0001). The median difference in LPPAI scores was 3.3 (2.56), reflecting a significant reduction in disease activity. Although 9 (12.2%) patients showed no change in LPPAI after treatment, the overall decrease in the LPPAI score is clinically relevant.

###  Coexisting Dermatological Diseases

 Coexisting dermatological diseases included: eczema: 4 (5.4%), alopecia areata: 3 (4.1%), atopic dermatitis: 6 (8.1%), psoriasis: 3 (4.1%), cutaneous lichen planus: 9 (12.2%), buttock folliculitis: 1 (1.4%), and vitiligo: 1 (1.4%). Medication history of patients included in this study in shown in Table 1.

**Table 1:** Prevalence of Medication Use in the Study Sample

	**Frequency (Percent)**
Prednisolone	10 (13.5)
Cyclosporine	13 (17.5)
MMF	2 (2.7)
Intralesional corticosteroid injection	5 (6.7)
Hydroxychloroquine	8 (10.8)
Baricitinib	3 (4.0)
Methotrexate	4 (5.4)

###  History of Autoimmune Diseases

 Patients had a history of the following autoimmune diseases:

 Autoimmune thyroiditis: 17 (23%), diabetes: 2 (2.7%), arthritis: 1 (1.4%).

###  Family History of Autoimmune Diseases

 A family history of autoimmune disease was present in 16 (21.6%) patients, including 15 (20.3%) with a family history of thyroiditis, and one (1.4%) with a family history of lichen planus.

###  Complications

 The complications associated with tofacitinib use were: Headache: 6 (8.1%), hyperlipidemia: 2 (2.7%), elevated liver enzymes: 4 (5.4%), nausea: 5 (6.8%), and high blood pressure: 3 (4.1%). The complications associated with Tofactinib consumption is shown in Table 2.

**Table 2:** Complications According to Demographic Characteristics

		**Headache**	**Hyperlipidemia**	**Elevated Liver Enzymes**	**Nausea**	**High Blood Pressure**	**Without Complications**
Gender	Female	5 (8.1%)	2 (3.2%)	4 (6.5%)	5 (8.1%)	3(4.8%)	43 (69.4%)
	Male	1 (8.3%)	0 (0%)	0 (0%)	0 (0%)	0(0%)	11 (91.7%)
Age (y)		47.8 ± 3.8	53 ± 1.4	50.5 ± 5.2	52 ± 3.1	52.6 ± 2.5	

###  Response to Treatment

 The response rate to tofacitinib was as follows:

- 9 (12.2%) patients did not respond.
- 16 (21.6%) patients responded within 1‒3 months.
- 18 (24.3%) patients responded within 3‒6 months.
- 25 (33.8%) patients responded within 6‒12 months.
- 6 (8.1%) patients responded within 12‒24 months.

## Discussion

 The effectiveness of tofacitinib treatment in patients with LPP was assessed in this study, revealing a significant improvement in disease activity based on LPPA. The reduction in the average LPPA score from 4.61 ± 1.26 before treatment to 1.73 ± 1.68 six months after treatment was significant (*P*-value = 0.0001), indicating the efficacy of tofacitinib in treatment of LPP. Our study included 74 patients, predominantly female (83.3%), with a mean age of 46.64 ± 8.05 years. The average age at diagnosis was 42.01 ± 7.95 years, and the average disease duration was 55.04 ± 37.52 months. These demographics are in line with the typical profile of LPP patients, who are often middle-aged women.

 The reduction in LPPAI scores aligns with previous studies on tofacitinib, a Janus kinase (JAK1,3) inhibitor, in treating autoimmune skin conditions. For instance, Mackay-Wiggan et al found that tofacitinib effectively treated alopecia areata, another autoimmune condition, with patients experiencing significant hair regrowth and reduced disease activity.^19^ Similarly, Craiglow and King reported significant improvements in patients with alopecia universalis treated with tofacitinib.^20^ The results of our study align with these findings, as 91.84% of our patients experienced a decrease in disease activity, with different response times: 21.62% within 1‒3 months, 24.32% within 3‒6 months, 33.78% within 6‒12 months, and 8.10% within 12‒24 months.

 In a case series by Jabbari et al, patients with LPP treated with tofacitinib exhibited significant improvement in both clinical symptoms and histopathological features.^21^ Our study supports these findings by demonstrating a significant reduction in disease activity. Furthermore, Damsky et al documented favorable outcomes in patients with various inflammatory skin conditions who were treated with tofacitinib, emphasizing its potential in the management of autoimmune skin disorders.^22^ Improvement of patients’ conditions in these studies highlights tofacitinib’s efficacy in treating disorders with pathophysiological mechanisms similar to LPP.

 The presence of comorbid dermatological conditions (e.g. eczema, alopecia areata, atopic dermatitis, psoriasis, cutaneous lichen planus) and autoimmune diseases (e.g. autoimmune thyroiditis, diabetes, arthritis) in our study is notable. A significant subset of patients (27%) had these comorbidities, highlighting the complexity of LPP and the need for screening for other autoimmune disorders. Papp et al also highlighted the importance of monitoring these comorbidities, particularly in patients with underlying health conditions.^23^

 In our study, tofacitinib demonstrated generally mild to moderate adverse effects, including headache (8.10%), hyperlipidemia (2.70%), elevated liver enzymes (5.40%), nausea (6.75%), and high blood pressure (4.15%). Interestingly, female patients exhibited a higher incidence of these side effects, suggesting potential gender-specific responses that warrant further investigation. These findings align with previous research; for instance, studies have reported similar adverse effects in rheumatoid arthritis patients treated with tofacitinib.^24^ Sandborn et al^25^ and Papp et al^26^ also underscored the need for monitoring liver function and lipid levels in patients with ulcerative colitis and plaque psoriasis, respectively. Additionally, our study found that patients previously treated with cyclosporine expressed satisfaction but switched to tofacitinib due to cyclosporine-associated complications like hypertension. This transition highlights the need to balance effectiveness and safety in long-term treatment plans for chronic conditions. Notably, one patient who was not satisfied with tofacitinib after six months was prescribed baricitinib, which led to a satisfactory outcome.^27^

 While this study provides valuable insights into the efficacy of tofacitinib for treating LPP, several limitations inherent in the study design should be considered. One significant limitation is the before-after design. This type of observational design lacks a control group, making it difficult to definitively attribute the observed improvements in disease activity solely to the treatment. Without a comparator group, there is a risk of regression to the mean.

 Additionally, the study does not account for time trends that may influence disease progression or treatment outcomes. We acknowledge that external factors that may have changed during the study period could confound the outcome. These limitations highlight the need for further investigations to validate the results. Future research should focus on identifying predictors of response to tofacitinib, exploring the long-term safety and efficacy of this treatment, potential long-term side effects, and comparing its effectiveness with other therapeutic options. Additionally, studies investigating the mechanisms underlying gender differences in adverse effects could provide valuable insights for optimizing patient management.

## Conclusion

 In conclusion, tofacitinib represents a promising treatment option for LPP, offering significant improvement in disease activity for the majority of patients. However, the variability in response and the incidence of adverse effects necessitate a personalized approach to treatment and accurate monitoring to ensure optimal outcomes.
